# Changes in Chemical Structure of Thermally Modified Spruce Wood Due to Decaying Fungi

**DOI:** 10.3390/jof8070739

**Published:** 2022-07-18

**Authors:** Zuzana Vidholdová, František Kačík, Ladislav Reinprecht, Viera Kučerová, Jana Luptáková

**Affiliations:** 1 Department of Wood Technology, Faculty of Wood Sciences and Technology, Technical University in Zvolen, T. G. Masaryka 24, SK-96001 Zvolen, Slovakia; ladislav.reinprecht@tuzvo.sk; 2 Department of Wood Chemistry and Chemical Technology, Faculty of Wood Sciences and Technology, Technical University in Zvolen, T. G. Masaryka 24, SK-96001 Zvolen, Slovakia; frantisek.kacik@tuzvo.sk (F.K.); viera.kucerova@tuzvo.sk (V.K.); janabercikova@azet.sk (J.L.)

**Keywords:** Norway spruce wood, chemical structure, thermal treatment, decaying fungi, *Serpula lacrymans*, *Trametes versicolor*

## Abstract

Fungi play a critical role in the decomposition of wood and wood-based products in use. The ability of decaying fungi to cause degradation of polysaccharides and lignin in the thermally modified Norway spruce (*Picea abies* L. Karst.) wood was examined with pure culture decomposition tests in laboratory conditions using the brown-rot fungus *Serpula lacrymans* (Schumacher ex Fries) S.F. Gray and white-rot fungus *Trametes versicolor* (Linnaeus ex Fries) Pilat. Spruce wood samples were primary thermally treated under atmospheric pressure at the temperatures of 100, 150, 200, 220, 240 and 260 °C during 1, 3 and 5 h, whereby larger losses in their mass, holocellulose, mannose and xylose were achieved at harder thermal regimes. Meanwhile, the holocellulose percent content reduced considerably, and the percent content of lignin increased sharply. Spruce wood thermally modified at and above 200 °C better resisted to brown-rot fungus *S. lacrymans* than the white-rot fungus *T. versicolor*. Due to the decay processes, the mass fractions of holocellulose, cellulose and hemicelluloses were lower in those spruce wood samples in which thermal degradation was more intensive, with achieving the highest mass loss values after thermal treatments, after which the decay attacks were poorer or even none with the minimal mass loss values due to action by the brown-rot fungus *S. lacrymans* and the white-rot fungus *T. versicolor*. The mannose and glucose percent content in thermally–fungally attacked spruce wood was intensive reduced, e.g., by 17% to 98% in wood after thermal treatments at temperature equal and above 200 °C.

## 1. Introduction

For the recycling of wood and wood-based products, it is important that the components, i.e., chemicals or other materials, added to wood in the different process stages do not prevent the use of the wood in the next cycle or prevent the return of ash to the natural life cycle [1]. From this view, the wood modification process based on thermal treatment in adding only energy (to creating heat) and water to the technology is a suitable and environmentally friendly process. Thermal treatment methods are knowledge-based processes. The improvement of important wood properties—such as hygroscopicity [2,3], dimensional stability [4], mechanical properties [5,6,7] and optical properties [8]—are functions of the process parameters, mainly of the temperature, humidity, presence of oxygen or presence of inert gases and time. Changes in various properties of wood are reflected in changes in the chemical structure of the wood of origin as a result of chemical reactions of the constituents of wood cells.

Currently, thermal treatment belongs to supplementary treatments, which are applied on site to improve the resistance of low- and non-durable wood species to attack by biological agents such as fungi, insects and animals. Resistance of thermally modified wood to different wood decaying fungi also depends on the thermal process parameters. Stamm et al. [9] reported the first systematic attempts to increase the resistance of thermowood to the white-rot fungus *Trametes serialis* by heating non-durable pine wood at temperatures between 140 °C and 320 °C. Buro [10] continued the work [9] by studying the thermal modification of pine and beech wood in different gaseous atmospheres. Thermal treatments affect the chemical structural factors of the wood required for the growth of fungi. According to studies of more researchers [11,12,13,14,15,16,17,18,19,20,21,22,23], decay resistance of thermally modified wood is related to the reduction in maximum moisture capacity of the cell walls due to changed and partly degraded wood polymers, mainly hemicelluloses, and due to modification of lignin and the creation of new linkages in the polysaccharide–lignin matrix of wood leading to a non-recognition of the latter by enzymes involved in fungal degradation of wood cell walls. In general, the resistance of wood products to rot is obviously better if their heat treatment is performed at higher temperatures and for longer times. At the same time, in the thermally modified woods the decay processes caused by brown-rot fungi are inhibited more as those ones caused by white-rot fungi.

Although several hundred fungi are responsible for the decay of wood in use in buildings and other wood base construction materials in temperate regions, the most common genera encountered are *Antrodia, Coniophora*, *Gloeophyllum*, *Meruliporia*, *Neolentinus* (formerly *Lentinus*), *Oligoporus*, and *Polyporus*. Of these genera, *Polyporus* is the only one that is a white-rot fungus. In both types of decay, the cell wall is degraded by fungal hyphae growing within the cell lumina, and cell wall degradation progresses from the lumen outward. Brown-rot fungi in nature serve as saprotrophs, decaying standing and downed wood in forests, contributing to forest regeneration and soil building processes or as parasites of trees usually occupying the roots or heartwood, acting as selective forces to propagate resistant individuals and a healthy forest population [24]. Much of the building construction in Europe is made of conifer wood. This is due to the fact that conifers account for 58.6% of the growing amount in European forests, while pine (29.6%) and spruce (23%) have the largest share [25]. However, the dominant and also an important species of conifers in forests in Slovakia is Norway spruce (*Picea abies*) (21.76% share in 2020 [26]). According to the EN 350 standard [27], spruce wood is only of low durability (durability class 4 (slightly durable) for mature wood and durability class 5 (not durable) for sapwood) and requires special protection against wood-destroying organisms, particularly when used in outdoor applications (with and without ground contact). Brown-rot fungi are extremely significant in the deterioration of wood in use, such as timbers, poles and in homes. Most of the wood decay in use in Europe is due to a single species of brown-rot fungus (*Coniophora puteana*). Additionally, *Serpula lacrymans* is a well-known fungus that causes dry rot of wood. Dry rot is a form of brown rot caused by certain fungi able to conduct water over long distances, and thus the wood appears dry when it is decayed. This fungus is regarded as the most damaging destroyer in buildings in temperate regions in Eurasia, North and South America and Oceania (Australia/New Zealand) [28,29]. It grows and spreads rapidly. It has low wood moisture requirements. It is able produce substantial quantities of water by breaking down the wood polysaccharide. The wood rapidly loses its strength after being attacked by a fungus [30]. Therefore, it is one of the test organisms recommended in the standard [31]. *T. versicolor* is a well-known white-rot fungal species. It produces simultaneous decay in hardwoods with reduced lignin and carbohydrate nearly at the same rate, with a slight preference for lignin [32]. Although white rot is less frequent in wooden construction, as it occurs most often in standing and felled many deciduous trees and some conifers, white-rot fungus is selected to compare it with brown-rot fungus. Wood progressively loses strength after being attacked by a fungus. As [33] stated, the flexural strength and compressive strength of Scots pine wood can decrease by up to 50% with 20% mass loss, respectively, with 30% mass loss.

This research determines the decay resistance of the Norway spruce wood thermally modified at different temperatures and times against selected brown-rot and white-rot fungi. In this regard, the aim of this study is to find the relationship between thermal inducted mass loss and fungal mass loss. However, degradation processes change the content and structure of particular wood polymers (cellulose, hemicelluloses and lignin) in the cell wall and interfere with their mutual interactions and arrangement. From this point of the view, its basic aim was to evaluate changes in the chemical structure of the thermally treated and following with the fungi-attacked wood samples. The chemistry of wood decay is a subject of importance because increased knowledge of the gradual chemical steps involved in the decomposition process can allow for more effective protection of natural wood and as well as thermally treated wood when used with a high risk of decomposition.

## 2. Materials and Methods

### 2.1. Spruce Wood

Samples of Norway spruce timber (*Picea abies* L. Karst.) with dimensions of 25 × 25 × 5 mm (longitudinal × tangential × radial) were used for the experiments.

### 2.2. Thermal Treatment of Spruce Wood

The wood samples were thermally treated in an oven (Memmert UNB 200, Fisher Scientific, Loughborough, UK), under atmospheric pressure at temperatures of 100 °C, 150 °C, 200 °C, 220 °C, 240 °C and 260 °C. The used temperatures were selected to examine the whole temperature interval generally used for heat treatment processes. Samples were placed in the oven preheated to the target temperature and kept there for 1, 3 or 5 h. Following, the samples were cooled in desiccators to 20 ± 2 °C and weighed with an accuracy of 0.001 g. Mass loss of each thermally treated sample was determined by the gravimetric method.

Samples, depending on the temperature of thermal treatment, were divided into several groups, and are further referred as “20” (control, reference), “100”, “150”, “200”, “220”, “240” and “260”.

### 2.3. Pure Fungal Culture Decomposition Test

The reference and thermally treated spruce wood samples were exposed to a pure fungal culture—either with the brown-rot fungus *Serpula lacrymans* (Schumacher ex Fries) S.F. Gray, strain BAM 87 (Bundesanstalt für Materialforshung und -prüfung, Berlin, Germany) or with the white-rot fungus *Trametes versicolor* (Linnaeus ex Fries) Pilat, strain BAM 116 (Bundesanstalt für Materialforschung und -prüfung, Berlin, Germany).

Fungal attacks were performed by a modified standard EN 113-2 [31], i.e., using smaller samples, another sterilization method and shorter incubation time—similarly with the work [6], and with the rapid mycological screening test [34]. In the vaccination box (Merci, Ferrara, Italy), two samples of the same type were placed into Petri dish with diameter of 100 mm on plastic mats under which a fungal mycelium was already grown up on 3–4 mm thick layer of the 4.5% malt agar soil (HiMedia, Laboratories Pvt. Ltd., Mumbia, India). The incubation process lasted 8 weeks at a temperature of 22 ± 2 °C and a relative air humidity of 70 ± 5%. Following, the samples were dried at 103 ± 2 °C to the oven-dried state, cooled in desiccators to 20 ± 2 °C and finally weighed with an accuracy of 0.001 g. The mass loss of each sample exposed to fungal attack was determined by the gravimetric method.

### 2.4. Chemical Analyses of Thermally and Thermally–Fungally Attacked Spruce Wood

The untreated (control, reference), thermally treated and thermally–fungally attacked spruce wood samples were mechanically disintegrated and milled to a particle size of 200–300 μm, using a POLYMIX PX-MFC 90D laboratory mill (Kinematica, Luzern, Switzerland), and wood particles were dried (4 h at 105 ± 2 °C).

According to ASTM D1107-21 [35], the extractives content in wood particles was determined in a Soxhlet apparatus with a mixture of absolute ethanol for analysis (Merck, Darmstadt, Germany) and toluene for analysis (Merck, Darmstadt, Germany) (1.0/0.427 *v*/*v*). The extraction lasted 8 h with six siphoning’s per hour.

Determination of the contents of structural carbohydrates (arabinose, galactose, mannose, xylose, glucose) and lignin in wood particles was carried out using high-performance liquid chromatography (HPLC) according to the National Renewable Energy Laboratory (NREL) analytical procedure [36].

Each sample was chemically analyzed in duplicate, and the results are presented as oven-dried wood percentages.

### 2.5. Statistical Evaluation

The software Microsoft 365^®^—Excel and MATLAB version R2021b (The MathWorks, Inc., Natick, MA, USA) was used to analyze the gathered data. The descriptive statistics deal with the basic statistical characteristics of the studied properties—the arithmetic mean and standard deviation. A regression model together with the coefficient of determination (*R*^2^) was used as a method of inductive statistics to evaluate the measured data. The coefficient of determination was evaluated at the 95% level of significance with the following scale: *R*^2^ ˂ 0.1—low degree of dependence, 0.1 ≤ *R*^2^ ˂ 0.25—slight degree of dependence, 0.25 ≤ *R*^2^ ˂ 0.5—significant degree of dependence, 0.5 ≤ *R*^2^ ˂ 0.8—high degree of dependence and 0.8 ≤ *R*^2^ ˂ 1—very high degree of dependence.

## 3. Results and Discussion

### 3.1. Mass Loss of Spruce Wood at Thermal Treatments

**Figure 1:**
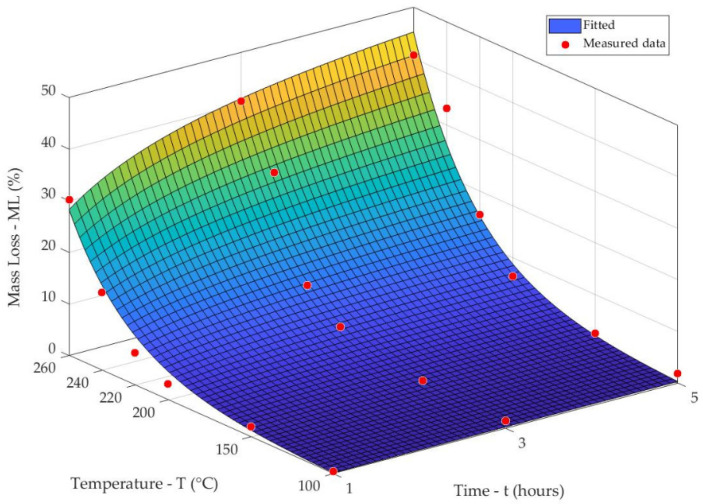
Mass loss values of the thermally treated spruce wood samples according to the time–temperature function: ML–T = (0.050·e^T/37.310^ − 0.378) · t/(0.874 + t); *R*^2^ = 0.957.

With increasing temperature levels during thermal treatment, a significant increase in mass loss was found (Figure 1).

From the 3D graph, it is evident that the increased mass losses of spruce wood were more influenced by the increased treatment temperatures as by the prolonged treatment times. The process of the thermal treatment (100–260 °C/1–5 h) of wood induced its mass losses in the range of 0.48–40.67%. The same results of mass loss could be obtained with different temperatures, depending on the treatment time. A noticeable mass loss at temperatures above 220 °C suggested there was intensive decomposition of the wood components in cell walls. Measured data were further compared to the three-dimensional analytical function, representing a combination of the hyperbolical and exponential functions proposed by studies [3,37]. The experimental results and the three-dimensional fitted surface show great agreement (*R*^2^ = 0.957), and the analytical model can be considered valid and applicable to the prediction of mass loss values caused by thermal treatments or environmental exposures of the elements of Norway spruce wood.

### 3.2. Mass Loss of Spruce Wood Caused by Decaying Fungi

**Figure 2:**
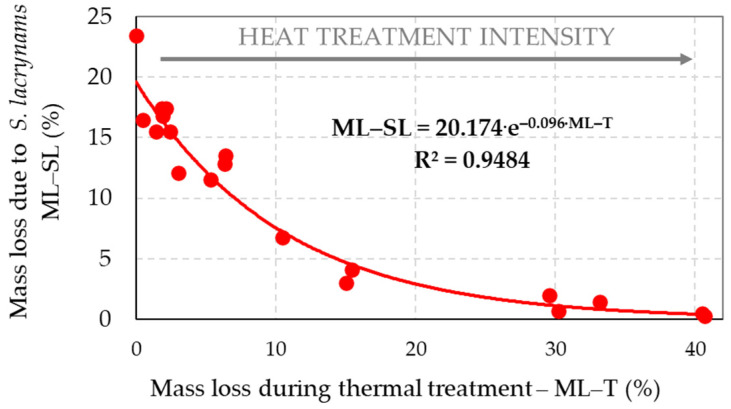
Correlation between the mass loss of wood resulting from its individual thermal treatments (ML–T) and the mass loss of wood caused by decaying fungi at 8-week mycological test by fungus brown-rot *S. lacrymans* (ML–SL).

**Figure 3:**
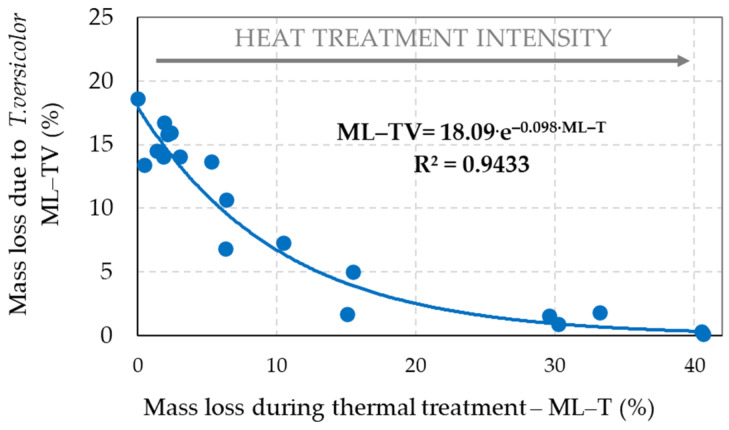
Correlation between the mass loss of wood resulting from its individual thermal treatments (ML–T) and the mass loss of wood caused by decaying fungi at 8–week mycological test by white-rot fungus *T. versicolor* (ML–TV).

The decay resistance of the reference and thermally treated spruce wood samples to the brown-rot fungus *S. lacrymans* and the white-rot fungus *T. versicolor* was evaluated on the basis of their mass losses (Figure 2 and Figure 3).

**Figure 4:**
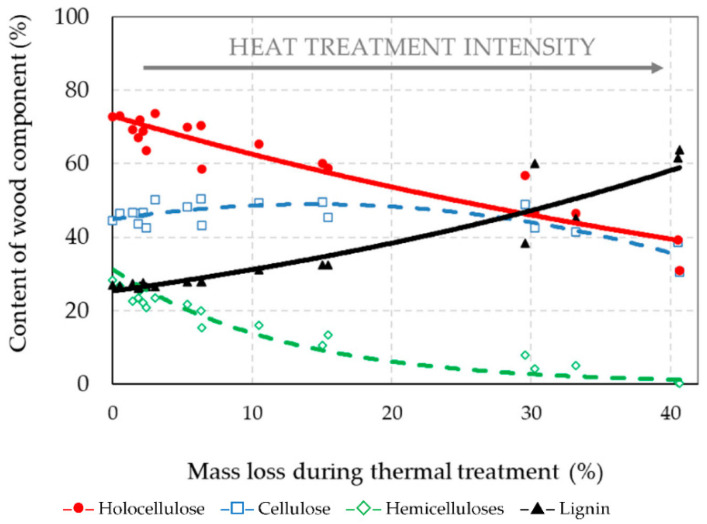
The percent content of wood components thermally treated spruce wood according to the mass loss during thermal treatment (Correlation equations are listed in Table 1).

Both used decaying fungi were capable of degrading the thermally treated wood. However, these fungi had a limited activity, explained by a change in the chemical composition of the wood during its thermal exposures (see point 3.3—Figure 4). Fungal attacks, recorded by mass loss, correlated well exponentially with the thermal treatment intensity (indicated by rising the mass loss “ML–T”), at which coefficients of determination *R*^2^ reached a very high degree of dependence (0.9484 at degradation by *S. lacrymans* and 0.9433 at degradation by *T. versicolor*). For a given temperature, increase of the treatment time improved gradually spruce wood durability against fungal attacks. Thermally treated spruce wood with mass losses of around 15% becomes totally resistant to decay attacks [17,38]. In the experiments carried out in this work, the increased resistance to decay of spruce wood (minimal values of ML–SL and ML–TV) strongly correlated with increased mass losses at thermal treatments (ML–T). It coincides well with results found by other authors [13,14,15,16,17,18,19,20,21,22,23]. The study [13] for beech wood (*Fagus sylvatica*) wood treated at 200 °C for 8 h in nitrogen atmosphere reported a mass loss of approximately 5% without significant effect on its subsequent decay; however, treatment at 240 °C evidently suppressed *T. versicolor* activity. These results are similar to study [39], where white-rot fungus *Picnoporus sanguineus* caused a reduction of between 15.7% and 82.4% in the weight loss in the 180–220 °C in the thermally treated samples from *Eucalyptus grandis*. The studies [15,18,19,20,21,22,23] indicated that decreased hygroscopic nature of wood and improved dimension stability of wood result in improved resistance against decay fungi, depending upon the intensity of treatment.

Heat treatment is generally less effective in protection against white-rot fungi compared to brown-rot fungi, which was shown at [40] for *Pleurotus ostreatus* and *Coniophora puteana* in Scotch pine (*Pinus sylvestris*) wood, at [41] for *T. versicolor* and *Rhodonia placenta* in thermally treated beech wood in presence of polyethylene glycol or at [42] for *T. versicolor* and *Oligoporus placentus* in Chir pine (*Pinus roxburghii*) wood.

According to the studies [11,12,13,14,15,16,17,18,19,20,21,22,23], decay resistance of thermally treated wood to be related to reduction in maximum moisture capacity of the cell walls due to reduced share of hydroxyl groups in hemicelluloses and lignin, partly degraded wood polymers—mainly hemicelluloses—and the creation of new linkages in the polysaccharide–lignin matrix of wood.

### 3.3. Chemical Structure of Thermally Treated Spruce Wood

The chemical compositions of the untreated (original) spruce wood and thermally treated spruce wood were determined using the NREL method. The chemical composition of the untreated spruce wood was the following: cellulose 44.46%, hemicelluloses 28.40% (while the proportion of selected hemicelluloses decreased in order: mannose > xylose > glucose ~ galactose ~ arabinose), and lignin 27.06%. With increasing mass loss at thermal treatments, a significantly rising change in the percent content of holocellulose (cellulose and hemicelluloses), and lignin in thermally treated materials was found (Figure 4, Table 1).

The amounts of holocellulose decreased in the thermally treated spruce wood, accompanied by increase in lignin. However, the proportion of cellulose increases slightly to the limit of approximately 15% mass loss (such same mass loss can be achieved after thermal treatment of spruce wood at 220 °C for 5 h or at 240 °C for 1 h) and then decreases again. The dependence of the cellulose content on mass loss during thermal treatment was expressed by the polynomial equation (Table 1). The coefficient of determination *R*^2^ was 0.678, which represents a high degree of dependence. Similar findings concerning the increase in cellulose content during heat treatment were obtained in the research [43].

**Figure 5:**
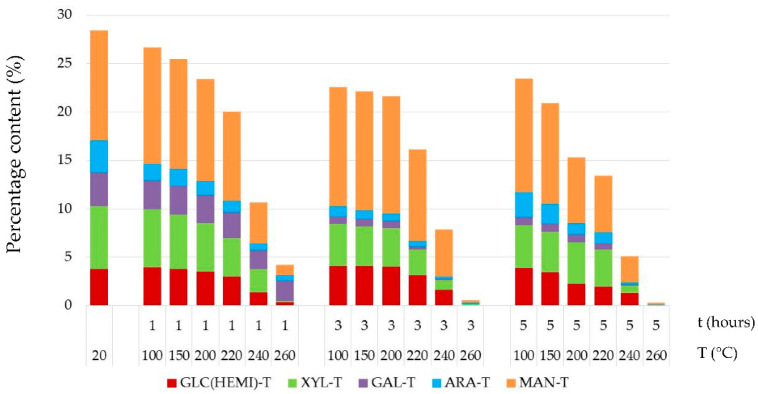
Percentage contents of the individual hemicelluloses in thermally treated wood. Note: ARA = arabinose, GAL = galactose, MAN = mannose, XYL = xylose and GLC = glucose.

Generally, hemicelluloses upon heat exposure are degraded faster than cellulose. Thermal treatments at temperatures equal and above 200 °C caused degradation of hemicelluloses of approximately 17.57–98.87%. From the point of the view of hemicelluloses present in spruce wood, in previous research works [44,45], the most extensive thermal degradation occurred in mannose, to a lesser extent in xylose and to the least extent in galactose. In our experiment, similar degradation order of individual hemicelluloses types was found “arabinose > mannose ~ glucose  >  xylose ~ galactose”, e.g., was found out at 15% mass loss of wood arabinose decreased by 85%, mannose by 70%, glucose by 67% and xylose and galactose by 40% (Figure 5). The percentage content of individual hemicellulose types in the thermally treated spruce wood had a mostly decreasing tendency until the application of a temperature of 220 °C, with mannose, xylose and glucose retaining the largest portion.

This finding was in good accordance with the study [46], where the mass loss of carbohydrates is illustrated as a function of the total mass loss in the thermally treated spruce samples. In thermally modified Scots pine sapwood (treatment temperature was 185, 200, 215 and 230 °C during 3 h), mannose and xylose were more stable than arabinose and galactose; however, the change in wood saccharides in hemicelluloses as a function of the mass loss by heat treatment showed a similar pattern [43]. In heat-treated oak wood (treatment temperature was 160, 180 and 210 °C according to Thermowood process), the least stable saccharides were galactose, arabinose and mannose, almost completely decomposed by thermal modification [45]. The study [45] also found out that using the Thermowood process at 210 °C the hemicelluloses in spruce wood dropped by 37.40% and in oak wood by 58.85%, which suggests that hemicelluloses in coniferous woods are thermally more stable than in deciduous woods, at which a higher temperature also reduces the amount of acetyl groups. Hemicelluloses generally have a lower thermal stability than cellulose, presumably due to their lack of crystallinity and lower degree of polymerization [5,46]. In our experiment, the most intensive thermal degradation of hemicelluloses occurred in the temperature range of 240–260 °C, mainly at the longest time of 5 h. Due to thermal treatment at 240 °C, the content of hemicelluloses decreased between 63% and 82%, while at 260 °C, they almost completely disappeared from the wood and their highest disintegration was found after 5 h of thermal treatment (Figure 5). The thermal degradation of hemicelluloses was the result of their depolymerization, the creation of internal ethers and some other rearrangement products and, finally, the formation of volatile compounds [5,8,47]. Release of acetic acid by degradation of hemicelluloses might have limited the colonization of fungus and consequently improved decay resistance of treated samples [11]. Transformation of hemicelluloses from hydrophilic and easily digestible to hydrophobic molecules during heat treatment was also one of the possible factors that contributed to lower weight loss of treated samples [47].

Another important indicator of the degradability of wood during heat treatment is the lignin content, as it is a very thermally stable polymer [8,41,42,46]. The amount of lignin in the thermally treated spruce wood increased with an increase in the intensity of the treatment, that is, as a result of degradation of polysaccharides (Figure 4). For example, after treatment at 260 °C for 5 h, it was 2.4 times higher compared to untreated spruce wood. These results are similar to [48], where the lignin content of samples increased with increasing temperature of treatment and extended time. The increase in lignin content during thermal modification is caused by the condensation of by-products that occurs by the degradation of hemicelluloses and amorphous cellulose, which become entrapped forming a repolymerization of lignin [11,49,50,51].

### 3.4. Chemical Structure of Thermally–Fungally Attacked Spruce Wood

**Figure 6:**
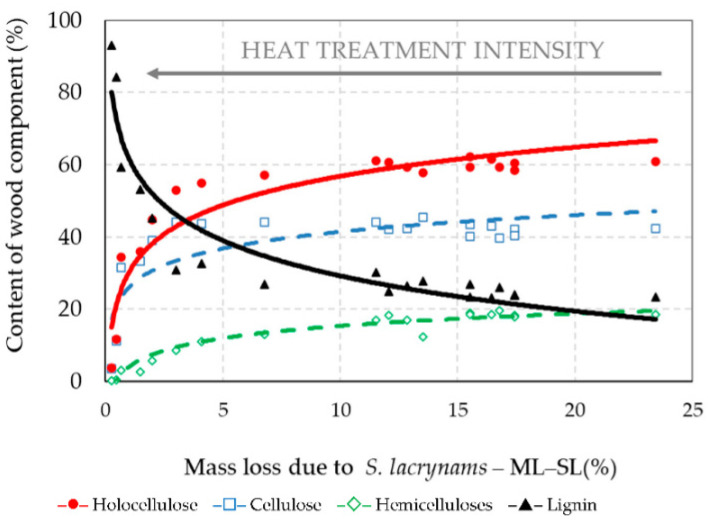
The percent content of wood components of the thermally–fungally attacked spruce wood according to the mass loss caused by *S. lacrymans* (Correlation equations are listed in Table 2).

**Figure 7:**
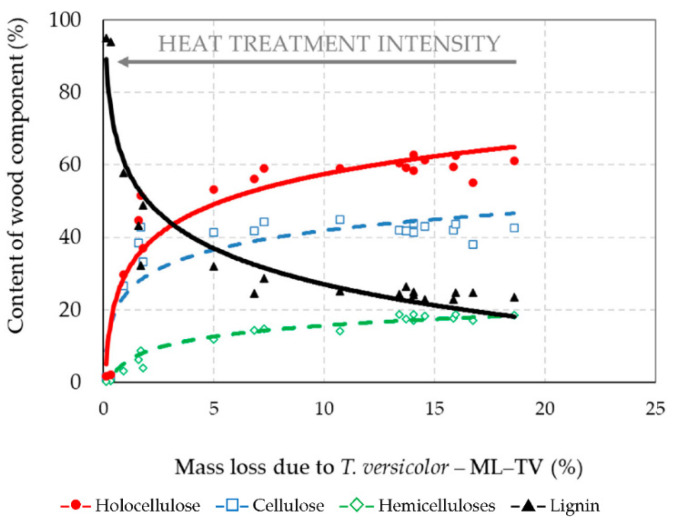
The percent content of wood components of the thermally–fungally attacked spruce wood according to the mass loss caused by *T. versicolor* (Correlation equations are listed in Table 3).

The chemical compositions of the thermally treated spruce wood samples after attacking by fungi *S. lacrymans* and *T. versicolor* determined according to the NREL technical report [36] are expressed by logarithmic correlations in relation to the mass losses in Figure 6 and Figure 7, and also in Table 2 and Table 3.

The amount of main chemical compounds in the thermally–fungally attacked wood is influenced by the mass loss caused by both decaying fungi. Brown-rot fungi are able to more rapidly and selectively degrade and extract the cellulose from wood than white-rot fungi. Lignin is, however, modified by the brown-rot fungi via demethylations and various oxidative reactions, including some cleavage of aromatic ring structures [24]. The results showed that due to the decay processes the mass fractions of holocellulose, cellulose and hemicelluloses were lower in the spruce wood samples for which thermal degradation was more intense with the achievement of the highest ML–T values, and the following, where the decay attacks were poorer or even none with the minimal ML–SL and ML–TV values (Figure 2 and Figure 3). The hemicelluloses content was very well correlated with the loss of mass of wood during its fungal decomposition, and the determination coefficients reached the level of a very high degree of dependence—*R*^2^ was 0.938 and 0.936. As we expected, hemicelluloses were the most bio-degradable component of wood, mainly when it was treated less intensively during thermal exposures.

In the case of cellulose, its content in the decayed thermally treated samples slowly increased from 38.03% to 45.41% in the range of mass loss of 5–23% due to its mainly crystalline structure.

**Figure 8:**
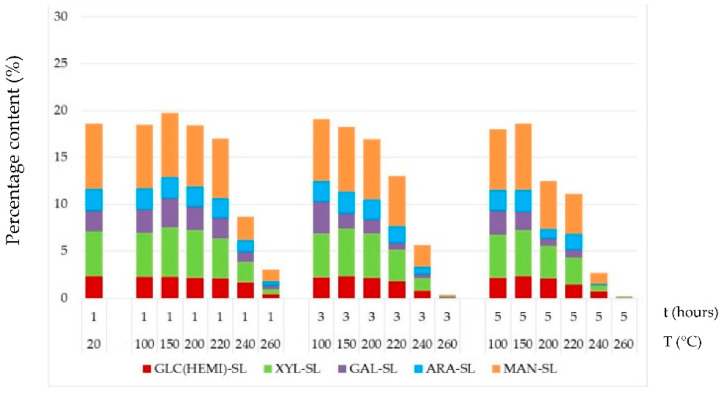
Percentage contents of the individual hemicelluloses in thermally–fungally attacked spruce wood by *S. lacrymans*. Note: ARA = arabinose, GAL = galactose, MAN = mannose, XYL = xylose and GLC = glucose.

**Figure 9:**
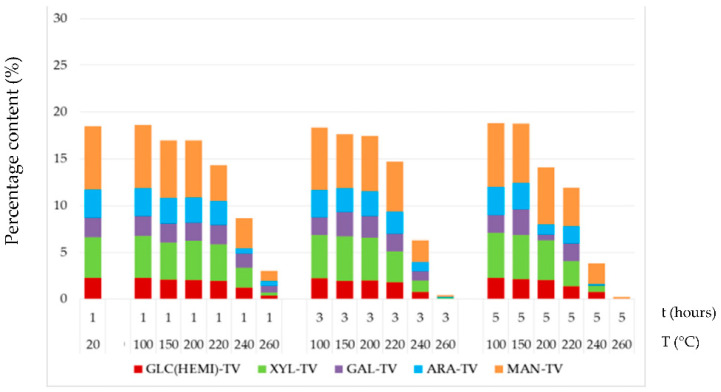
Percentage contents of the individual hemicelluloses in thermally–fungally attacked spruce wood by *T. versicolor*. Note: ARA = arabinose, GAL = galactose, MAN = mannose, XYL = xylose and GLC = glucose.

Fungal attack in thermally treated spruce wood samples caused a reduction in mannose and glucose content (Figure 8 and Figure 9) compared to decay-free samples (Figure 5). Both fungi degraded these saccharides to the similar extent. Their content decreased by 17% to 98% in wood after thermal treatments at temperature equal and above 200 °C. However, this finding was not observed in the study [43] in thermally modified Scots pine sapwood (treatment temperature was 185, 200, 215 and 230 °C for 3 h), which was attacked by brown-rot fungus *Rhodonia placenta* over 16 weeks. This difference might be explained by the fact that the influence of various factors, such as wood species, enzymatic activities of fungi, conditions of sterilization and mycological test, influence of high humidity environment during the decomposition process, test duration and so on are different. Xylose, as the second most abundant of non-glucosic carbohydrate in spruce wood, was more intensively degraded by the white-rot fungus *T. versicolor* than by the brown-rot fungus *S. lacrymans*. However, in the thermally treated spruce, its degradation was milder, mainly by *T. versicolor*. The portion of galactose and arabinose in thermally–fungally attacked wood varied minimally.

As shown earlier [11,12,13,14,15,16,17,18,19,20,21,22,23,38,39,40,41,42,52], the higher mass loss of wood in the heating process, the more biologically durable the heat-treated wood. One of the generally accepted reasons for the higher decay resistance in heat-treated wood is due to the loss of hemicelluloses polymers in the cell walls [11,13,15]. The innovative point of view to explain the fungal degradation behavior of thermally modified wood was presented in study [51]. As indicated in this study, pseudo-lignin formation could play an important role in the various fungal degradation performance of hydrothermally modified wood and fungi species. Pseudo-lignin has a structure similar to lignin, so it could possibly behave similarly in fungal degradation, namely, poor resistance against white-rot fungi.

## 4. Conclusions

- Different temperatures and times during the thermal treatments of spruce wood, from 100 °C to 260 °C/1–5 h, had varied effects on its chemical composition and decay resistance.
- Thermal treatments at temperatures equal to and above 200 °C caused high mass losses of spruce wood, from 3.04% to 40.67%, and great degradation of hemicelluloses, about 17.57–98.87%. Meanwhile, the holocellulose percent content reduced considerably and the percent content of lignin increased sharply.
- The decrease of holocellulose in spruce wood thermally modified at stronger thermal conditions leaded to the reduction of its bio-attacks by the brown-rot fungus *S. lacrymans* and the white-rot fungus *T. versicolor*, i.e., the decay resistance of such thermally modified woods can be significantly improved, more evidently against brown-rot fungi.
- Due to the fungal attacks by the brown-rot fungus *S. lacrymans* and the white-rot fungus *T. versicolor* the holocellulose, cellulose and hemicelluloses mass fractions were lower in those spruce wood samples in which primordial thermal degradation was highly intensive with achieving very high mass losses. On the contrary, the decay attacks were more intensive in those spruce samples that were not thermally treated, or their primordial thermal degradation was poorer with the minimal mass losses or even none.
- The mannose and glucose percent content in the thermally–fungally attacked spruce wood was mainly reduced, e.g., by 17% up to 98% in samples primordially treated at temperatures equal and above 200 °C.

## Figures and Tables

**Table 1:** Correlations between the content of wood components and the mass loss of the Norway spruce wood samples caused by thermal treatments.

Wood Component	Percentage Content of Wood Component = f (Mass Loss (ML–T))	Coefficient of Determination (*R*^2^)
Holocellulose	HOLO–CEL = 72.99·e^−0.015·(ML–T)^	*R*^2^ = 0.868
Cellulose	CEL = 44.89 + 0.574·(ML–T) − 0.020·(ML–T)^2^	*R*^2^ = 0.678
Hemicelluloses	HEMI–CEL = 31.26·e^−0.081·(ML–T)^	*R*^2^ = 0.941
Lignin	LIGNIN = 25.34·e^0.0208·(ML–T)^	*R*^2^ = 0.900

**Table 2:** Correlations between the percent content of wood components and the mass loss of the Norway spruce wood samples during fungal attack by *S. lacrymans*.

Wood Component	Percentage Content of Wood Component = f (Mass Loss (ML–SL))	Coefficient of Determination (*R*^2^)
Holocellulose	HOLO–CEL = 30.24 + 11.56·ln(ML–SL)	*R*^2^ = 0.880
Cellulose	CEL = 26.10 + 6.67·ln(ML–SL)	*R*^2^ = 0.672
Hemicelluloses	HEMI–CEL = 4.14 + 4.89·ln(ML–SL)	*R*^2^ = 0.938
Lignin	LIGNIN = 61.74 − 14.11·ln(ML–SL)	*R*^2^ = 0.885

**Table 3:** Correlations between the percent content of wood components and the mass loss of the Norway spruce wood samples during fungal attack by *T. versicolor*.

Wood Component	Percentage Content of Wood Component = f (Mass Loss (ML–TV))	Coefficient of Determination (*R*^2^)
Holocellulose	HOLO–CEL = 29.68 + 12.07·ln(ML–TV)	*R*^2^ = 0.886
Cellulose	CEL = 24.17 + 7.68·ln(ML–TV)	*R*^2^ = 0.743
Hemicelluloses	HEMI–CEL = 5.50 + 4.99·ln(ML–TV)	*R*^2^ = 0.936
Lignin	LIGNIN = 60.00 − 14.32·ln(ML–TV)	*R*^2^ = 0.878

## Data Availability

Not applicable.
